# Supplementary material for: Inconsistent Provider Testing Practices for Congenital Cytomegalovirus: Missed Diagnoses and Missed Opportunities
Source: Int J Neonatal Screen. 2022 Nov 14;8(4):60. doi: 10.3390/ijns8040060 (PMC9680485; doi:10.3390/ijns8040060)
Supplement: Supplementary file 1 [file IJNS-08-00060-s001.zip › IJNS-1787666-supplementary.pdf]

International Journal of *Neonatal Screening*

MDPI

Supplementary

Inconsistent Provider Testing Practices for Congenital Cytomegalovirus: Missed Diagnoses and Missed Opportunities

Kate L. Wilson [1,2,*], Kimi Shah [2] and Megan H. Pesch [2,3,*]

Table S1. Laboratory tests used to identify infants tested for congenital CMV at a quaternary health system in the United States from 2014-2019.

Keyword	Order/Result Name in DataDirect [11]	Order, Result/ LIONC? (as noted in DataDirect)	CPT Code	Include or Exclude	Reason/Notes
Cytomegalovirus	CYTOMEGALOVIRUS CULTURE [Order: CMVCULT]	Order	87254 or 87252	Exclude (order not result)	Culture of urine/CSF/serum can be used for infant CMV testing
Cytomegalovirus	Cytomegalovirus by Rapid PCR [Order: LCCMV	Order	87254 or 87252	Exclude (order not result)	
Cytomegalovirus	Cytomegalovirus DNA, Qual [Order: PCRCV]	Order	87496	Exclude (order not result)	
Cytomegalovirus	Cytomegalovirus DNA, Quantitation [Order: OCMV]	Order	87497	Exclude (order not result)	
Cytomegalovirus	Cytomegalovirus IgG, Qual [Order: CMVG]	Order	86644	Exclude (order not result)	Not a sensitive or specific test, but can be used in infants
Cytomegalovirus	Cytomegalovirus IgM, Qual [Order: CMVM]	Result	86645	Include	
Cytomegalovirus	Cytomegalovirus by Rapid PCR [Result: LCCMV]	Result	87254 or 87252	Include	Urine PCR send-out
Cytomegalovirus	CYTOMEGALOVIRUS CULTURE[Result: CMVCULT]	Result	87254 or 87252	Include	
Cytomegalovirus	Cytomegalovirus DNA, Qual [Result: PCRCV]	Result	87496	Include	
Cytomegalovirus	Cytomegalovirus [Presence] in Unspecified specimen by Organism specific culture [LOINC: 5838-8]	Result	87254 or 87252	Include	
Cytomegalovirus	Cytomegalovirus DNA [#/volume] (viral load) in Bone marrow by Probe and target amplification method [LOINC: 49350-2]	Result	87496	Exclude	Not used for congenital CMV
Cytomegalovirus	Cytomegalovirus DNA [Units/volume] (viral load) in Unspecified specimen by Probe and target amplification method [LOINC: 34720-3]	Result	87496	Include	
Cytomegalovirus	Cytomegalovirus IgG Ab [Interpretation] in Serum [LOINC: 20475-0]	Result	86644	Include	
Cytomegalovirus	Cytomegalovirus IgG Ab [Presence] in Serum by Immuno-	Result	86644	Include	

	Assay [LOINC: 13949-3]				
Cytomegalovirus	Cytomegalovirus IgG Ab [Units/volume] in Serum or Plasma by Immunoassay [LOINC: 5124-3]	Result		Include	
Cytomegalovirus	Cytomegalovirus IgM Ab [Presence] in Serum by Immunofluorescence [LOINC: 49539-0]	Result	86645	Include	
Cytomegalovirus	Cytomegalovirus IgM Ab [Presence] in Serum or Plasma by Immunoassay [LOINC: 24119-0]	Result	86645	Include	
CMV	CMV ANTIGENEMIA CMV AVR, Plasma [Order: VCAVR]	Order	87496	Exclude (order not result)	
CMV	CMV DNA qPCR [Order: VCMV]	Order	87497	Exclude (order not result)	
CMV	CMV DNA qPCR [Order: VCMVU]	Order	87497	Exclude (order not result)	
CMV	CMV DNA qPCR, Blood [Order: VBCMV]	Order	87497	Exclude (order not result)	
CMV	CMV DNA, QUANTITATION; PCR [Order: QCMV]	Order	87497	Exclude (order not result)	
CMV	CMV DNA, Quantitative PCR EX [Order: VCMV EX]	Order	87497	Exclude (order not result)	
CMV	CMV DNA, QUANTITATIVE, ENDPT [Order: QCMVEP]	Order	87497	Exclude (order not result)	
CMV	CMV IGG ANTIBODY BY EIA [Order: CMVGE]	Order	86644	Exclude (order not result)	
CMV	CMV IGG ANTIBODY TITER BY IFA [Order: CMVGT]	Order	86644	Exclude (order not result)	
CMV	CMV IGM ANTIBODY TITER [Order: CMVMT]	Order	86644	Exclude (order not result)	
CMV	CMV PCR Qual (tissue) [Order: VCMQL]	Order	87496	Exclude (order not result)	Tissue pathology not used in cCMV except placenta which would be in in the mother's chart
CMV	CMV qPCR (aqueous fluid) [Order: VCMAQ]	Order	87497	Exclude (order not result)	Aqueous fluid not used to test for cCMV
CMV	CMV qPCR (BAL) 5500 [Order: VCMBL]	Order	87497	Exclude (order not result)	Bronchiolavage fluid not used to test for cCMV
CMV	CMV qPCR (Bone Marrow) 5500 [Order: VCMBM]	Order	87497	Exclude (order not result)	Bone Marrow not used to test for cCMV
CMV	CMV qPCR (vitreous fluid) [Order: VCMVF]	Order	87497	Exclude (order not result)	Vitreous (eye) fluid not used to test for cCMV
CMV	CMV qPCR Quant, Fresh Tissue [Order: VCMVT]	Order	87497	Exclude (order not result)	Fresh tissue (skin, organs etc.) not used in cCMVexcept placenta which would be in the mother's chart
CMV	CMV qPCR, CSF [Order: VCMVC]	Order	87497	Exclude (order not result)	
CMV	CMV Quant by PCR, Blood Spot [Order: UWCBS]	Order	87497	Exclude (order not result)	
CMV	CMV Resistance: Maribavir, Ganciclovir, Foscarnet, Cidofovir [Order: VCMR4]	Order	87910	Exclude (order not result)	This test used for immuno-suppressed patients on long-term

					antivirals not to diagnose cCMV
CMV	CMV Titers EX [Order: CMV EX]	Order	86644	Exclude (order not result)	
CMV	COMPREHENSIVE RESP VIRAL CULT [Order: CRCMV]	Order	87507	Exclude (order not result)	Used for upper respira- tory infections, never concern for congenital infection
CMV	Cytomegalovirus by Rapid PCR [Order: LCCMV]	Order	87254 or 87252	Exclude (order not result)	
CMV	CYTOMEGALOVIRUS CULTURE [Order: CMVCULT]	Order	87254 or 87252	Exclude (order not result)	
CMV	Cytomegalovirus DNA, Quantitation [Order: OCMV]	Order	87497	Exclude (order not result)	
CMV	Cytomegalovirus IgG, Qual [Order: CMVG]	Order	86644	Exclude (order not result)	
CMV	Cytomegalovirus IgM, Qual [Order: CMVM]	Order	86645	Exclude (order not result)	
CMV	Donor Anti-CMV [Order: DCMV]	Order		Exclude (order not result)	
CMV	External Lab CMV DNA, Quantitative PCR (QUEST) [Order: QVCMV]	Order	87497	Exclude (order not result)	
CMV	External Lab CMV IgG and IgM Quant Titers [Order: XCMVQ]	Order	86644	Exclude (order not result)	
CMV	External Lab CMV Qual PCR (copies/mL) [Order: XVC2Q]	Order	87496	Exclude (order not result)	
CMV	External Lab CMV Qual PCR (IU/mL) [Order: XVCMQ]	Order	87496	Exclude (order not result)	
CMV	External Lab CMV Quantitative (cop- ies/mL) [Order: XVCM2]	Order	87497	Exclude (order not result)	
CMV	External Lab CMV Titers (QUEST) [Or- der: QCMVG]	Order		Exclude (order not result)	
CMV	SHELL VIAL-CMV BY IFA [Order: SVCMV]	Order	87252; 87254	Exclude (order not result)	Type of culture
CMV	VIRAL CULTURE EXCLUDING CMV [Order: VCULT]	Order	87254 or 87252	Exclude (order not result)	
CMV	VIRAL CULTURE INCLUDING CMV [Order: CVCULT]	Order	87254 or 87252	Exclude (order not result)	
CMV	VIRAL RESP CULTURE- GENERAL/CMV [Order: RCMV]	Order	87254 or 87252	Exclude (order not result)	
CMV	CMV ANTIGENEMIA [Result: CMVAG]	Result	87496	Include	
CMV	CMV DNA qPCR [Result: VCMV]	Result	87497	Include	
CMV	CMV DNA qPCR Result [Result: VCMVR]	Result	87497	Include	
CMV	CMV DNA qPCR, Blood [Result: VBCMV]	Result	87497	Include	
CMV	CMV DNA, QN PCR EX (QUEST) [Re- sult: X0217]	Result	87497	Include	
CMV	CMV DNA, QN REAL TIME PCR EX (QUEST) [Result: X0118]	Result	87497	Include	
CMV	CMV DNA, QUANTITATION; PCR [Result: QCMV]	Result	87497	Include	

CMV	CMV DNA, Quantitative PCR (copies/mL) EX [Result: X2VCM]	Result	87497	Include	
CMV	CMV DNA, Quantitative PCR EX [Result: VCMV EX]	Result	87497	Include	
CMV	CMV DNA, Quantitative PCR EX [Result: XXVCM]	Result	87497	Include	
CMV	CMV DNA, QUANTITATIVE, ENDPT [Result: QCMVEP]	Result	87497	Include	
CMV	CMV IGG ANTIBODY BY EIA [Result: CMVGE]	Result	86644	Include	
CMV	CMV IgG Antibody, Qualitative [Result: CMVGI]	Result	86644	Include	
CMV	CMV IGG EX [Result: CMV IGG EX]	Result	86644	Include	
CMV	CMV IgG EX [Result: XCMVG]	Result	86644	Include	
CMV	CMV IgG EX (QUEST) [Result: X0160]	Result	86644	Include	
CMV	CMV IgG Titer Quant EX [Result: XXCMG]	Result	86644	Include	
CMV	CMV IGM ANTIBODY TITER [Result: CMVMT]	Result	86645	Include	
CMV	CMV IgM Antibody, Qualitative [Result: CMVMI]	Result	86645	Include	
CMV	CMV IGM EX [Result: CMV IGM EX]	Result	86645	Include	
CMV	CMV IgM EX [Result: XCMVM]	Result	86645	Include	
CMV	CMV IgM Titer Quant EX [Result: XXCMM]	Result	86645	Include	
CMV	CMV PCR Qual, Tissue [Result: Z1115]	Result	87497	Exclude	Tissue not used in cCMV except if placenta which would be in mother's chart
CMV	CMV qPCR (aqueous fluid) 5500 [Result: Z1116]	Result	87497	Exclude	Aqueous fluid not used to test for cCMV
CMV	CMV qPCR (Bone Marrow) 5500 [Result: Z1044]	Result	87497	Exclude	Bone marrow not used to test for cCMV
CMV	CMV qPCR (vitreous fluid) 5500 [Result: Z1119]	Result	87497	Exclude	Vitreous (eye) fluid not used to test for cCMV
CMV	CMV qPCR Quant Result, Tissue [Result: Z0992]	Result	87497	Exclude	Tissue not used in cCMV except if placenta which would be in mother's chart
CMV	CMV qPCR Quant, Type of Tissue [Result: Z0993]	Result	87497	Exclude	Tissue not used in cCMV except if placenta which would be in mother's chart
CMV	CMV QPCR Result [Result: Z2357]	Result	87497	Include	
CMV	CMV Qual PCR (copies/mL) EX [Result: XXVC2]	Result	87497	Include	
CMV	CMV Qual PCR (IU/mL) EX [Result: XXCMV]	Result	87497	Include	

CMV	CMV Result, CSF [Result: Z0910]	Result	87497 or 87254	Include	
CMV	CMV Result, Urine [Result: Z0909]	Result	87497 or 87254	Include	
CMV	COMPREHENSIVE RESP VIRAL CULT [Result: CRCMV]	Result	87633	Exclude	Test used for upper respir-atory infections not for cCMV
CMV	Cytomegalovirus by Rapid PCR [Re-sult: LCCMV]	Result	87254 or 87252	Include	
CMV	CYTOMEGALOVIRUS CULTURE [Result: CMVCULT]	Result	87254 or 87252	Include	
CMV	QCMV Log10 Result: [Result: QCMVRL]	Result		Include	
CMV	QCMV M2000 Interpretation Result: [Result: QCMVRI]	Result	87497	Include	Interpretation always paired with quantitative or qualitative results
CMV	QCMV Result: [Result: QCMVR]	Result		Include	
CMV	SHELL VIAL-CMV BY IFA [Result: SVCMV]	Result	87252; 87254	Include	Type of culture
CMV	VIRAL CULTURE EXCLUDING CMV [Result: VCULT]	Result	87254 or 87252	Include	
CMV	VIRAL CULTURE INCLUDING CMV [Result: CVCULT]	Result	87254 or 87252	Include	
CMV	VIRAL RESP CULTURE-GENERAL/CMV [Result: RCMV]	Result	87254 or 87252	Exclude	Respiratory cultures used for respiratory infections, not cCMV test

Table S2. Data elements and definitions.

					Data from clinical medical record, laboratory records, EHR only applicable for CMV test cohort in the days prior to CMV testing (not after)	Data from EHR ≤ 31 days
**Variable name**	**Type of variable**	**Units**	**Response options/ formatting**	**Data source ***	**Further definition/where located in chart for cohort tested for CMV**	**Further definition/where located in chart for larger cohort NOT tested for CMV (if different)**
**Subject ID**	Whole number	NA	#########	NA	Indirect identifiers created by DataDirect [11].	
**DOB**	Date	NA	MM/DD/YYYY	0	Discrete data from EHR by DataDirect [11].	
**Sex**	Categorical	NA	0=Male, 1=Female, 2=Unknown	0	Unknown if not apparent or "intersex". Discrete data from EHR by DataDirect [11].	
**GA**	Continuous	Weeks ^{days}	WW^{D} (days = X/7 fraction)	2	Discrete data from EHR for all inborn infants. Data manually abstracted by reviewing the birth/delivery or transfer of care notes for outborn infants in the CMV test cohort.	Only able to ascertain for inborn infants in this group via data pull from EHR.
**Delivery**	Binomial	NA	0=Vaginal, 1=C-Section	2	Discrete data from EHR for all inborn infants. Data manually abstracted by reviewing the birth/delivery or transfer of care notes for outborn infants in the CMV test cohort.	Only able to ascertain for inborn infants in this group via data pull from EHR.
**Maternal Age**	Whole number	Years	##	2	Maternal age at delivery. Discrete data from EHR for all inborn infants. Data manually abstracted by reviewing the birth/delivery or transfer of care notes for outborn infants in the CMV test cohort.	Only able to ascertain for inborn infants in this group via data pull from EHR.
**Maternal Parity**	Whole number	Live born children	#	2	Number of live born children at time of subject infant's birth (counting that infant), range of 0 (if stillborn first child) to X. Discrete data from EHR for all inborn infants. Data manually abstracted by reviewing the birth/delivery or transfer of care notes for outborn infants in the CMV test cohort.	Only able to ascertain for inborn infants in this group via data pull from EHR.
**Baby Race**	Descriptive categorical	NA	0=White, 1=Black, 2=Asian 3=Other, 4=Unknown	0	Race as identified in the EHR.. "Other" includes Native American, pacific islander, Native Alaskan etc. If baby had two or more races listed, the program defaults to selecting the first race listed. Biracial was not an option. Unknown if not listed in discrete field of record.	
**Baby Ethnicity**	Categorical	NA	0=Hispanic, 1=Non-Hispanic, 2=Unknown	0	Ethnicity as identified in the EHR. Unknown if not listed in discrete field of record.	
**Baby Insurance**	Categorical	NA	0=Public, 1=Private/ Commercial/ Other	0	Primary insurance type as listed in the EHR. Public insurance defined as primary insurance being Medicaid (having Medicaid secondary insurance to a primary private/commercial plan not counted as Medicaid). Other if self-pay or unknown.	
**Delivery Location**	Binomial	NA	0=Inborn, 1=Outborn	0	Location where infant was born. Inborn is at study institution. Outborn is anywhere else.	Subjects with birth records at study institution considered inborn, all others considered outborn.
**Test Date**	Date	NA	MM/DD/YYYY	1	Date that the test for CMV was collected (e.g., specimen collected, which could be different from the date the test was ordered or the results returned). If multiple CMV tests ordered, include only data for the first test resulted). Data found in the laboratory results and/or orders tabs of the EHR. Must see the laboratory test and result, not just read a descriptive sentence about it in a clinical note.	

**Test Type**	Categorical		0=CMV PCR, 1 = CMV IgG/IgM, 2= CMV Cx, 3=Other	1	Type of test that was first ordered to test for CMV . If multiple CMV tests ordered, include only data for the first test resulted. Data found in the laboratory results and/or orders tabs of the EHR. Must see the laboratory test and result, not just read a descriptive sentence about it in a clinical note. CMV PCR = polymerase chain reaction test (or send out NAAT) of any fluid (serum/plasma, dried blood spot, urine, CSF, and hypothetically saliva although not available at this institution), CMV IgG/IgM = any CMV antibody testing performed on the infant. Maternal antibody testing is not to be counted. CMV culture = viral culture of bodily fluid (urine, CSF, or serum/plasma) that includes CMV. Does not include viral cultures of bone marrow or respiratory secretions. 3=Other includes anything else. Please include free text description of this test.	NA
**CMV Status**	Binomial	NA	0=Negative, 1=Positive	1	Results of the CMV test qualitatively - was CMV isolated or not? Do not include units/ML or other quantitative information here. Considered positive if CMV identified in the test and no notes included about poor sample, or ambiguous.	NA
**CMV Type**	Binomial	NA	0=cCMV, 1=pnCMV	1	Type of CMV that the clinical team was evaluating the infant for congenital (cCMV) or postnatal (pnCMV) [12]. Data found by reviewing the clinical notes surrounding the dates of the testing, and if not clear, then any subsequent consultation notes (e.g., from Pediatric Infectious Disease specialists) if ambiguity were to arise.	NA
**Birth Wt**	Number	kg	#.###	2	Birthweight as recorded in EHR, double check with discrete field. Weight on day of life "0" not DOL 1.	Only able to ascertain for inborn infants in this group via data pull from EHR.
**Birth Wt%ile (WHO)***	Percentile	NA	##%ile	Calc'd BW, sex, GA by-WHO	Birthweight by percentile if ≥37 weeks using WHO growth chart [12]. Discrete data from EHR for all inborn infants. Data manually abstracted by reviewing the birth/ delivery or transfer of care notes for outborn infants in the CMV test cohort.	Only able to calculate for inborn infants in this group via data pull from EHR.

**Birth Wt%ile (Fenton)***	Percentile	NA	##%ile	Calc'd BW, sex, GA by Fenton	Birthweight by percentile if <37 weeks using Fenton growth chart [13]. Discrete data from EHR for all inborn infants. Data manually abstracted by reviewing the birth/ delivery or transfer of care notes for outborn infants in the CMV test cohort.	Only able to calculate for inborn infants in this group via data pull from EHR.
**SGA**	Binary	NA	0=Yes, 1=No, 2=Unknown		Birth measurement defined as <10%ile for gestational age/sex [12, 13].	
**Birth Length**	Number	cm	##	2	Discrete data from EHR for all inborn infants. Data manually abstracted by reviewing the birth/delivery or transfer of care notes for outborn infants in the CMV test cohort.	Only able to calculate for inborn infants in this group via data pull from EHR.

**Birth HC**	Number	cm	##	2	Discrete data from EHR for all inborn infants. Data manually abstracted by reviewing the birth/delivery or transfer of care notes for outborn infants in the CMV test cohort. Note if reviewing the growth chart, and there are several measurements in the first days of life, with the first measurement being an outlier (much smaller than others), this may not represent true microcephaly but rather molding. In this case, consider the HC measurement that is in line with following measurements in the chart, not the outlier. Do not chose HC measurements taken outside first week of life.	Only able to calculate for inborn infants in this group via data pull from EHR.
**Birth HC%ile (WHO)**	Percentile	NA	##%ile	Calc'd HC, sex, GA by WHO	Head circumference by percentile if ≥37 weeks using WHO growth chart [12]. Discrete data from EHR for all inborn infants. Data manually abstracted by reviewing the birth/ delivery or transfer of care notes for outborn infants in the CMV test cohort.	Only able to calculate for inborn infants in this group via data pull from EHR.
**Birth HC%ile (Fenton)**	Percentile	NA	##%ile	Calc'd HC, sex, GA by Fenton	Head circumference by percentile if <37 weeks using Fenton growth chart [13]. Discrete data from EHR for all inborn infants. Data manually abstracted by reviewing the birth/ delivery or transfer of care notes for outborn infants in the CMV test cohort.	Only able to calculate for inborn infants in this group via data pull from EHR.
**Microcephaly**	Binary	NA	0=Present, 1=Absent/unknown	2	Birth measurement defined as <10%ile for gestational age/sex [12,13].	

**Petechiae**	Binary	NA	0=Present, 1=Absent/unknown	2	Review of clinical notes, and especially physical exams within the notes leading up to the CMV test. Presence of petechiae, petechial rash, purple pinpoint rash, purpuric rash in physical exam and/or assessment and plan.	Presence vs absence of diagnosis of petechiae in medical record. Defined as ICD-9-CM 772.6, 782.7; ICD-10-CM P54.5 [14,15]
**Jaundice**	Binary	NA	0=Present, 1=Absent/unknown	2	Review of clinical notes, and especially physical exams within the notes leading up to the CMV test. Presence of jaundice in physical exam and/or elevated total bilirubin.	Presence vs absence of diagnosis of jaundice in medical record. Defined as ICD-9-CM 774; ICD-10-CM P58.x or P59.x [14]
**Seizures**	Binary	NA	0=Present, 1=Absent/unknown	2	Review of clinical notes (including consultations from Pediatric Neurology or EEG monitoring reports) for epilepsy or seizures. Do not count "concern for seizures" if this has been ruled out. But count "seizures" if this appears to be a sign that is believed to be true at the time of testing (even if eventually ruled out).	Presence vs absence of diagnosis of seizures (and related terms) in medical record. Defined as 345.X and 780.3, 780.x, or 779.0 [16]
**Splenomegaly**	Binary	NA	0=Present, 1=Absent/unknown	2	Review of clinical notes, especially physical exams within the notes leading up to the CMV test. Presence of splenomegaly, enlarged spleen, HSM in physical exam finding and/or assessment and plan.	Presence vs absence of diagnosis of splenomegaly in medical record. Defined as ICD- 9-CM 759.0, 789.2; ICD-10-CM Q89.09, R16.1, or R16.2 [14,15
**Hepatomegaly**	Binary	NA	0=Present, 1=Absent/unknown	2	Review of clinical notes, especially physical exams within the notes leading up to the CMV test. Presence of hepatomegaly, enlarged liver, liver span below costal	Presence vs absence of diagnosis of hepatomegaly in medical record. Defined as ICD- 9-CM 751.69, 789.1; ICD-10-CM Q44.7, R16.0, R16.2, or B25.1 [14,15]

					margin, HSM in physical exam finding and/or assessment and plan.	
**Thrombo-cytopenia (<150)**	Binary	NA	0=Present, 1=Absent/unknown	2	Presence of at least one visualized laboratory result of platelets <150,000 K/uL. Need to see lab results, not just mentioned in the notes.	Presence vs absence of diagnosis of thrombocytopenia in medical record. Defined as ICD-9-CM 776.1, 776.2, 287.3–287.5; ICD-10-CM P60, P61.0, or D69.4x-D69.6x [14,15]. Or one laboratory value of platelets <150,000 K/uL (LOINC 777-3) [3]
**Direct Hyper-bilirubinemia (DB>3)**	Binary	NA	0=Present, 1=Absent/unknown	2	Presence of at least one visualized laboratory result of direct or conjugated bilirubin level of >3.0 mg/dL. Need to see lab results, not just mentioned in the notes.	Presence vs absence of diagnosis of direct or conjugated hyperbilirubinemia in medical record or one laboratory value of direct/conjugated bilirubin >3 mg/dL [3]
**Transaminitis (AST>80)**	Binary	NA	0=Present, 1=Absent/unknown	2	Presence of at least one visualized laboratory result of AST >80 IU/L. Need to see lab results, not just mentioned in the notes.	Presence vs absence of diagnosis of transaminitis in medical record or one laboratory value of AST >80 IU/L [3]
**Transaminitis (ALT >100)**	Binary	NA	0=Present, 1=Absent/unknown	2	Presence of at least one visualized laboratory result of ALT >100 IU/L. Need to see lab results, not just mentioned in the notes.	Presence vs absence of diagnosis of transaminitis in medical record or one laboratory value of ALT>100 IU/L [3]
**Referred Newborn Hearing Screen**	Binary	NA	0=Present, 1=Absent/unknown	2	Review of clinical notes or audiograms from birth hospital. Presence of "refer" hearing screen in one or both ears on audiogram. Because hearing screens can be repeated in the same admission, count the "final hearing screen" prior to the CMV test.	Presence vs absence of diagnosis of failed newborn hearing screen in medical record. Defined as ICD-9 CMV 794.15, ICD-10 P09 [17,18]. Or encounter for hearing examination following failed hearing screening in medical record. Defined as r94.120, R94.8 z01.118, or Z01.110 [17,18]
**Intracranial Abnormality**	Binary	NA	0=Present, 1=Absent/unknown	2	Review of cranial imaging reports obtained within ≤ 31 days. Presence of abnormalities including ventriculomegaly, intracerebral calcifications, periventricular echogenicity, cortical or cerebellar malformations [3].	Presence vs absence of diagnosis of intracranial abnormalities in medical record. Defined as ICD- 9-CM 348.89, 742.2–742.4, 793.0; ICD-10-CM G91.xx, G93.89, G93.9, Q03.xx, Q04.xx, or R90.82 [14]
**Retinitis**	Binary	NA	0=Present, 1=Absent/unknown	2	Review of clinical notes from Ophthalmology. Presence of chorioretinitis on exam. Do not count retinopathy of prematurity or ocular issues attributable to other obvious causes. If Ophthalmology not consulted, mark as unknown.	Presence vs absence of diagnosis of chorioretinal/retinitis in medical record. Defined as ICD-9-CM 363.0–3; ICD-10-CM H30.0x, H30.1x, H30.89x, H30.9x, or H31.00x [14]
**Prenatal concern for cCMV**	Binary	NA	0=Present, 1=Absent/unknown	1	Review of clinical notes, especially initial History and Physical Exam note which references maternal, pregnancy, and prenatal history if available (linked maternal/neonatal charts in the EHR). Direct access to maternal chart unavailable. Presence of prenatal concerns includes maternal CMV serologies, abnormal ultrasound findings, abnormal amniocentesis results, and/or obstetric and/or maternal fetal medicine notes.	NA. Data only collected for infants tested for CMV.
**Unit for Admission s/p Birth**	Binary	NA	0=NICU, 1=PCTU, 2=NBN, 3=PICU, 4=Home	1	Clinical unit where the infant was admitted and cared for immediately after birth.	NA. Data only collected for infants tested for CMV.

**Unit that Obtained Test**	Binary	NA	0=NICU, 1=PCTU, 2=NBN, 3=PICU, 4=Peds	1	Clinical unit and/or service where the infant was cared for by healthcare provider who ordered CMV testing. Unit may differ from the admission unit after birth.	NA. Data only collected for infants tested for CMV.
**Cardiac Baby**	Binary	NA	0=Yes, 1=No	1	Review of clinical notes. Presence of critical congenital cardiac defects necessitating admission and care in the Pediatric Cardiac Intensive Care Unit (PCTU), whose primary care team was Pediatric Cardiology, or whether testing for CMV was ordered by a Pediatric Cardiologist as part of a heart transplant evaluation. This does not include a preterm infant in the NICU with a cardiac complication whose care is not mainly directed by Pediatric Cardiology.	NA. Data only collected for infants tested for CMV.
**Screening Indication**	Free text	NA	Free text	1	Review of the clinical notes leading up to the CMV test. Presence of the documented testing indication(s) that prompted the provider to order CMV test. Indication(s) defined as the reason(s) the provider specifically documented as to why CMV test was ordered. If problem-based plan, then include the "problem" under which the CMV test is ordered. If systems-based plan, then include the "system" under which the CMV test is ordered. Do not include any signs that are not specifically documented as raising concerns for CMV or prompting testing (i.e. clinical findings which may differ from indications for testing).	NA. Data only collected for infants tested for CMV.

*** 0=Pulled by DataDirect** or Oracle/Clarity from institution EHR (discrete fields), **1=Manually** identified by review of the clinical record (CMV test cohort only),**2=Data pull for inborn infants in larger cohort, manual review** of clinical record of CMV test cohort

Abbreviations: ID- identification; DOB-date of birth; EHR-electronic health record; GA-gestational age; CMV-cytomegalovirus; NA-not applicable; PCR-polymerase chain reaction; Cx- culture; DOL-day of life; wt- weight; HC- head circumference; kg- kilograms; cm-centimeters; HSM-hepatosplenomegaly; s/p-status post; NICU- Neonatal Intensive Care Unit; NBN- Newborn Nursery; PCTU-Pediatric Cardiac Thoracic Unit; PICU- Pediatric Intensive Care Unit; Peds- General Pediatrics.
